# Urinary Tract Infections in Relation to Bladder Emptying in Patients with Spinal Cord Injury

**DOI:** 10.3390/jcm13133898

**Published:** 2024-07-02

**Authors:** Sasa Milicevic, Aleksandra Sekulic, Dejan Nikolic, Snezana Tomasevic-Todorovic, Konstansa Lazarevic, Svetislav Pelemis, Masa Petrovic, Sindi Z. Mitrovic

**Affiliations:** 1 Faculty of Medicine, University of Pristina, 38220 Kosovska Mitrovica, Serbia; 2 Clinic for Rehabilitation “Dr Miroslav Zotovic”, 11000 Belgrade, Serbia; 3 Faculty of Medicine, University of Belgrade, 11000 Belgrade, Serbia; 4 Department of Physical Medicine and Rehabilitation, University Children’s Hospital, 11000 Belgrade, Serbia; 5 Faculty of Medicine, University of Novi Sad, 21000 Novi Sad, Serbia; 6 Clinic for Medical Rehabilitation, Clinical Center of Vojvodina, 21000 Novi Sad, Serbia; 7 Department for Biomedical Science, State University of Novi Pazar, 36300 Novi Pazar, Serbia; 8 Clinic for Otorhinolaryngology and Maxillofacial Surgery, Clinical Center of Serbia, 11000 Belgrade, Serbia; 9 Institute for Cardiovascular Diseases “Dedinje”, 11000 Belgrade, Serbia

**Keywords:** spinal cord injuries, rehabilitation, urinary tract infections

## Abstract

**Background/Objectives**: Spinal cord injuries are debilitating conditions with significant impacts on physical function and patient quality of life. The high incidence of urinary tract infections in these patients can be attributed to neurogenic bladder—a major complication of spinal cord injuries. The aim of this study is to investigate the incidence of urinary tract infections in patients with spinal cord injuries and their relation to the method of bladder emptying. **Methods**: A retrospective analysis on 560 patients admitted for rehabilitation at the Clinic for Rehabilitation “Dr Miroslav Zotovic” from December 2009 to January 2023 was conducted. Patients over 18 years old who were inpatients for longer than 30 days without any symptoms of urinary tract infection on admission were included. Patient demographics, injury details, and bladder emptying methods were recorded. **Results**: In our study, 402 (71.8%) participants developed a urinary tract infection during their rehabilitation. Urinary tract infections were significantly more common in patients with traumatic spinal cord injuries, lower ASIA scores, and thoracic-level injuries. The highest incidence of urinary tract infections was observed in intermittent self-catheterization patients (62.7%), with *Escherichia coli* and *Proteus mirabilis* being the most frequently isolated pathogens. **Conclusions**: The method of bladder emptying significantly impacted the incidence of urinary tract infection in patients with spinal cord injuries. Despite guidelines favoring intermittent catheterization, this study found it to be associated with the highest infection rates. These findings suggest a need for personalized bladder management strategies to reduce the risk of urinary tract infections and improve outcomes for spinal cord injury patients.

## 1. Introduction

Spinal cord injuries (SCIs) represent a significant medical condition that can have a profound impact on an individual’s physical function, quality of life, and healthcare resource utilization [1,2,3,4]. Population-based studies have noted an estimated annual incidence of SCI ranging from 11.5 to 53.4 cases per one million inhabitants, noting an increasing incidence and global burden over the years [1,5]. Injuries to the spinal cord can result from traumatic events, such as motor vehicle accidents, falls, and acts of violence, or from non-traumatic causes, like infections, tumors, and degenerative diseases. [6] Notably, the severity of SCIs can vary widely, from partial impairment, affecting motor or sensory functions, to complete paralysis, defined by a total loss of muscle function and sensation below the level of injury.

The rehabilitation period is long term, and thus secondary complications are common and can ultimately influence overall patient quality of life, employability, rehospitalization, and the continuum of care [2,3,4]. One of the most significant complications following SCI is the loss of genitourinary and gastrointestinal function [2]. The disruption of normal neural pathways responsible for bladder control often leads to neurogenic bladder—a condition characterized by impaired bladder sensation, storage, and emptying [7]. This not only complicates SCI management but ultimately poses an increased risk of the development of urinary tract infections (UTIs), one of the most common complications in patients with SCI [8].

Long term, the recurrent nature of UTIs, particularly in the scope of the SCI population, can unfortunately progress to more serious complications such as acute kidney injury, urologic tumor development, hydronephrosis, sepsis, or even renal failure, further complicating clinical management and negatively impacting quality of life [9,10]. Therefore, the management of neurogenic bladder dysfunction remains a pivotal aspect in the rehabilitation of patients with SCI, especially when taking into consideration the significant impact it has on increasing morbidity and mortality due to renal failure [11,12]. Notably, the method of bladder emptying emerges as a key predictive factor for the incidence of UTIs, with evidence suggesting an elevated frequency of symptomatic UTIs in patients employing transurethral indwelling catheters [8,13,14]. Despite several clinical guidelines advocating for intermittent catheterization as a preferable alternative to diminish the risk of infections, the Consortium for Spinal Cord Medicine acknowledges the existence of mixed evidence, thereby refraining from endorsing one method unequivocally over the other [14]. Additional predictive factors for UTIs have been identified, including male gender, level of injury, completeness of the lesion, presence of detrusor sphincter dyssynergia, and vesicoureteral reflux, each contributing to the complex risk profile for urinary complications in this patient population [15,16,17,18].

The link between SCI and the increased risk of UTIs can be attributed to several factors, including the use of indwelling or intermittent catheterization for bladder management, incomplete bladder emptying, and changes in urinary flora. Catheterization, especially long-term indwelling catheter use, is a well-documented risk factor for UTIs due to the potential for introducing bacteria into the bladder [10]. Moreover, the altered bladder dynamics and stasis contribute to an environment conducive to bacterial growth and infection. Consequently, the minute management of UTIs in this patient population requires a multifaceted approach including antibiotic therapy, adjustments in bladder management strategies, and in some cases, surgical interventions may need to be employed.

The aim of our study was to investigate the incidence of UTIs in patients with SCIs and their relation to the method of bladder emptying.

## 2. Materials and Methods

### 2.1. Study Design and Participants

This retrospective study analyzed data from 560 patients with SCI admitted for rehabilitation at the Clinic for Rehabilitation “Dr Miroslav Zotovic” in Belgrade, Serbia, from December 2009 to January 2023. The inclusion criteria were as follows: at least 18 years old, inpatient for longer than 30 days, absence of any symptoms of UTI on admission, and having a SCI within the past month. Patients who were discharged or transferred to another center due to other injuries or complications within the first 30 days (i.e., stroke, deep vein thrombosis, pneumonia, cardiovascular complications, myocardial infarction) were excluded from the study. Factors that could potentially exacerbate the risk for developing UTIs, such as age, sex, type of injury, presence of other associated injuries, polytrauma, completeness of injury (ASIA Score), injury etiology, neurological level of injury, length of stay, method of bladder emptying, and presence and type of neurogenic bladder, were noted on admission. Prior to analysis, all patient-identifying information was anonymized. This study was conducted in alignment with the principles of the Declaration of Helsinki. Given the retrospective nature of this study, the requirement for individual informed consent was exempted. The study was approved by the Ethics Committee of the Clinic for Rehabilitation “Dr Miroslav Zotovic” (No. 03-1882/2013).

### 2.2. Data Collection and Evaluation

Upon admission, we collected detailed medical histories from all patients, including demographics (age, sex) and type of trauma. Each patient underwent a comprehensive physical examination to assess pain, motor, and sensory deficits related to SCI, incorporating the American Spinal Injury Association Impairment Scale (AIS) for determining the neurological level and completeness of the lesion. These medical records facilitated the classification of the mechanism of injury, SCI level, injury completeness, associated injuries, and length of stay. Furthermore, patients underwent radiological imaging that provided additional insights into their condition.

### 2.3. Catheterization Protocols

Patients with indwelling catheters underwent catheter changes every 2–4 weeks, in line with standard clinical practice. For patients utilizing intermittent catheterization and self-catheterization, the following protocols were followed:

Intermittent Catheterization: Clean, staff-assisted catheterizations were performed at regular intervals, typically every 4–6 h. Staff members received specialized training to minimize the risk of infection during these procedures.

Self-Catheterization: Patients were thoroughly trained by medical staff according to the institution’s training practices. The training process included education on proper techniques, supervised practice sessions, and continuous monitoring. Initially, patients were instructed to perform the procedure on their own while being closely supervised by medical staff. Once patients demonstrated proficiency and confidence in the procedure, medical staff continued to supervise them at least once daily to ensure the correct technique was being followed. Patients were instructed to perform intermittent catheterization every 4–6 h, adhering to the standards of clinical practice.

### 2.4. UTI Diagnosis and Treatment

Patients exhibiting clinical symptoms indicative of a UTI, such as fever, had a urine culture taken. A positive urine culture, defined as the presence of ≥10^5^ CFU/mL of a uropathogen, confirmed the diagnosis of UTI. Urine cultures included antibiograms to determine the antibiotic sensitivity of the isolated pathogens. Antibiotic treatment was then tailored based on the urine culture results and the presence of a body temperature exceeding 38 °C. Following antibiotic therapy, all subjects received a control urine culture test to evaluate the treatment’s effectiveness.

### 2.5. Statistical Methods

Depending on the type of variables and their distribution’s normality, which was assessed by the Kolmogorov–Smirnov test and by a visual analysis of histogram of frequencies and Q-Q plots, data were described using n (%), arithmetic mean ± standard deviation, or median (range: min–max). The following statistical hypothesis testing methods were applied: the *t*-test for comparing means of normally distributed continuous data, the Mann–Whitney test for non-normally distributed continuous or ordinal data, the chi-square test for nominal variables with expected frequencies greater than five, and Fisher’s exact probability test for nominal variables with expected frequencies of five or less. Logistic regression analysis was employed to examine the relationship between binary outcomes and potential predictors. Statistical hypotheses were tested at a significance level of 0.05, ensuring that results with a *p*-value less than this threshold were considered statistically significant. Data were analyzed using SPSS (Statistical Package for the Social Sciences) 29.0 (IBM Corp., Armonk, NY, USA).

## 3. Results

Our study population comprised 405 (72.3%) males and 155 (28.0%) females with an average age of 46.2 ± 16.7. Of the 560 patients included in our study, 402 (71.8%) patients had a UTI during their inpatient rehabilitation, with a predominance in the younger population, and there was no statistically significant difference between genders (Table 1). Further analysis revealed a higher incidence of UTIs in patients with traumatic SCIs compared to those with non-traumatic SCIs—68.2% versus 31.8%, respectively, reaching a level of statistical significance (Chi-square = 44.688; *p* < 0.001). Additionally, UTIs were more common in patients who had a lower ASIA score on admission (*p* < 0.001). When analyzing patients by neurological level of injury, a statistically significant difference was observed between groups, where UTIs were the most common in patients with thoracic-level injuries (Chi-square = 10.285, *p* = 0.006). Notably, the average length of stay was also longer for patients with UTIs (*p* < 0.001).

With regard to the modality of injury, in patients with traumatic SCIs, the most common injury was a fall from a height (46.9%), followed by traffic accidents (39.7%), jumping in water (7.2%), and firearm-related trauma (6.2%); however, there was no statistically significant relationship between the modality of injury and the incidence of UTIs in traumatic SCI patients (Fisher’s exact test, *p* = 0.139). Conversely, a statistically significant difference was observed between the modality of injury and the incidence of UTIs in patients with non-traumatic SCI (Chi-square = 24.237, *p* < 0.001). The most common causes of non-traumatic SCI were tumors (36.9%), followed by myelopathy (32.9%), infectious diseases (11.6%), vascular diseases (9.8%), polyradiculoneuritis (6.2%), and pathological fractures (2.7%).

The frequency of UTIs was significantly associated with the manner of bladder emptying differed (Table 2). UTIs occurred most often in patients using intermittent self-catheterization (62.7%), followed by patients using intermittent catheterization by medical staff (17.2%), patients with an indwelling catheter (7.7%), and patients relying on reflex emptying (6.5%), and they were least common in patients with spontaneous emptying (6%).

Among the patients who had a UTI during their rehabilitation period, 30.5% of patients had only one infection. The remainder of the patients who had a UTI suffered from multiple infections, with the number of infections ranging from 1 to 11.

The majority of these patients presented with a singular bacterial isolate (37.6%), although the spectrum of isolated bacteria ranged from one to seven distinct species. The most commonly isolated bacteria included *Escherichia coli* (52.1%), *Proteus mirabilis* (40.3%), *Pseudomonas* (35.8%), and *Klebsiella* (30.3%).

Upon further stratifying the data by the method of bladder management, significant variances in bacterial profiles were observed. Patients employing intermittent catheterization exhibited a bacterial distribution characterized predominantly by *Escherichia coli* (56.5%), with *Acinetobacter* species (13.0%) and a collective of other bacterial species (4.3%). Individuals engaging in intermittent self-catheterization predominantly encountered *Escherichia coli* (46.4%), *Proteus mirabilis* (33.8%), and a minor representation of other bacterial species (3.6%). Patients with indwelling catheters demonstrated a predisposition toward infection by specific bacterial species, notably *Proteus mirabilis* (51.6%), *Pseudomonas* (51.6%), *Klebsiella* (41.9%), and *Enterococcus* (19.4%). Distinctly, patients with reflex emptying methods had UTIs that were more frequently associated with infections by *Providencia* (28.6%) and *Morganella* (17.9%), in comparison with other methodologies of bladder management.

A significant portion of the study cohort (71.9%) were administered antibiotics based on their urine cultures. The majority of these patients (39.4%) were prescribed a single antibiotic to manage their UTI, with aminoglycoside antibiotics being the predominant class of antimicrobial agents utilized (35.0%).

Further analysis revealed that patients with UTIs most commonly exhibited hyper-reflexive bladder dysfunction (49.8%). We observed a significant difference in the frequency of UTIs when the cohort was further stratified by type of bladder dysfunction (Chi-square = 289.254; *p* < 0.001) (Table 3). 

In our analysis utilizing a multiple logistic regression model, we included seven predictors that were statistically significant in simple logistic regression models and predictors that were considered potentially significant for the occurrence of UTIs during rehabilitation, as suggested by previous research (Table 4). The complete model, including all predictors, was statistically significant (Chi-square = 299.220; DF = 9; *p* < 0.001) and explained 60% of the variance of the dependent variable. Due to multicollinearity with the bladder impairment variable, the mode of bladder emptying was excluded from the multivariate model. There is no significant multicollinearity between the other predictors in the model.

In the multiple logistic regression model, bladder impairment was identified as a statistically significant predictor of UTIs during rehabilitation. Specifically, when controlling for all other factors in the model, having a hyper-reflexive bladder increases the odds of developing an infection by over 60 times, and a hypotonic bladder increases it by nearly 16 times, compared to patients without bladder impairment. These findings are controlled for all other factors in the model.

## 4. Discussion

In our study, a significant portion of patients (72.3%) had a UTI during their inpatient rehabilitation, further corroborating the narrative established by the existing literature that cites UTIs as one of the most common secondary complications in SCI patients. Notably, in our cohort, we observed a greater number of UTIs in patients with traumatic SCIs compared to non-traumatic SCIs. Such a parallel reinforces the importance of understanding that the nature of the SCI—traumatic versus non-traumatic—may significantly influence the risk of developing a UTI. As observed in the multiple logistic regression model of our cohort, associated injuries emerged as a notable predictor, further supporting this finding.

With specific attention paid to the method of bladder emptying, we observed the highest frequency of UTIs in intermittent self-catheterization patients. This highlights the potential role of microbial colonization and the frequency of catheterization in contributing to this increased risk. Repeated catheterization can introduce bacteria into the urinary tract, which may lead to infections, particularly if proper aseptic techniques are not strictly followed. Moreover, the increased manipulation of the urethra and bladder may predispose these patients to colonization by uropathogens. Furthermore, prolonged hospitalization may also predispose patients to a higher risk of UTI due to factors such as increased exposure to nosocomial pathogens and decreased mobility.

To mitigate these potential risks, it is essential to provide comprehensive patient education and training. Patients should be thoroughly instructed on proper catheterization techniques, including hand hygiene, the use of sterile equipment, and the correct procedure for catheter insertion and removal. Healthcare providers should also regularly review and reinforce these practices during follow-up visits.

Additionally, developing individualized catheterization schedules and considering alternative bladder management strategies for patients at higher risk of UTIs could further reduce infection rates. Regular monitoring and early intervention at the first signs of infection are also critical components of a comprehensive infection control strategy. By addressing these factors, we can improve clinical outcomes and quality of life for patients with spinal cord injuries.

This observation partially challenges existing guidelines set forth by the Consortium for Spinal Cord Medicine that have previously reported mixed evidence regarding the superiority of catheterization techniques [14]. Furthermore, a recent study conducted as a part of a Dutch multicenter research program highlighted that clean intermittent catheterization was the primary bladder-emptying method (42.6%), followed by condom catheter drainage (11.3%), indwelling suprapubic catheterization (11.3%), and voluntary bladder reflex triggering (11.0%) [19]. Additionally, research conducted at a specialized SCI rehabilitation center in Switzerland from 2013 to 2017 demonstrated that catheter users had a consistently higher adjusted incidence rate for UTIs than those employing spontaneous emptying, identifying the bladder emptying method as the primary risk factor for UTIs in SCI patients [20]. Our findings further call for the need for a nuanced approach to bladder management, taking into account patient-specific risks and lifestyle considerations.

Moreover, our findings also contribute to the growing body of evidence on the bacterial landscape of UTIs in the SCI population. Similar to other studies, we revealed that the most commonly isolated pathogens in our cohort were *Escherichia coli* (52.1%) followed by *Proteus mirabilis* (40.3%), *Pseudomonas* (35.8%), and *Klebsiella* (30.3%) [10,18,21,22,23]. As noted in our study, the predominant class of antimicrobial agents used were aminoglycosides, further reflecting current trends in clinical practice that aim to balance efficacy and concerns of resistance.

Furthermore, our study identified a statistically significant association between lower ASIA scores at admission and increased UTI risk, suggesting that greater injury severity may exacerbate susceptibility to infections [24,25,26,27,28]. The results of our research further go on to support the findings of multiple studies that posit that patients with severe SCIs have impaired immune responses, which in turn could contribute to the reason behind our findings. Similar to other studies, we observed the highest frequency in patients with thoracic-level injuries [18,21,29]. The linkage between neurogenic bladder dysfunction types and UTI risk, particularly the marked risk elevation in patients with hyper-reflexive and hypotonic bladders, merits further investigation.

Future research should aim to further dissect the intricate relationship between bladder management methods and UTI risk, exploring the efficacy of various catheterization techniques in different SCI patient demographics. Additionally, longitudinal studies examining the long-term impact of UTI management on patient quality of life and rehabilitation success are warranted. By building upon the foundational insights provided by our study and others in the field, the clinical community can better tailor interventions to the nuanced needs of the SCI population, ultimately advancing patient care and outcomes.

### Limitations

While our study offers significant insights into the incidence of UTIs in patients with SCIs and their relation to the method of bladder emptying, several limitations should be considered when interpreting the results. First, the retrospective nature of the study may introduce inherent biases, including potential selection bias. This could affect the representativeness of our sample and the generalizability of our findings.

We have addressed these limitations by implementing several mitigation strategies. To reduce selection bias, we employed rigorous inclusion and exclusion criteria and ensured a diverse sample population.

Despite these measures, the aforementioned limitations should be taken into account when interpreting the study results. Future prospective studies with larger sample sizes and more robust methodologies are recommended to confirm and extend our findings.

## 5. Conclusions

Our study highlights the significant role of bladder management methods in the prevalence of UTIs among SCI patients, further emphasizing the need for personalized bladder management strategies. Despite guidelines, our findings indicate that intermittent self-catheterization, the most common method, is associated with the highest UTI rates, suggesting that a one-size-fits-all approach may not be optimal for SCI patients. As a result, our study calls for a shift towards more adaptable clinical guidelines that can accommodate the diverse needs of SCI patients by reducing the risk of UTIs, improving patient quality of life, and ultimately fostering better patient outcomes in SCI rehabilitation.

## Figures and Tables

**Table 1:** Study population characteristics.

	UTI		
	Yes (n = 402)	No (n = 158)	*p*-Value
**Age**	45 ± 16.9	51 ± 15.2	**<0.001 ^a^**
**Gender (male)**	296 (73.6%)	109 (69.0%)	0.269 ^b^
**Type of Injury**			
Traumatic	274 (68.2%)	59 (37.3%)	**<0.001 ^b^**
Non-traumatic	128 (31.8%)	99 (62.7%)	
**Associated Injuries (yes)**	110 (27.4%)	22 (13.9%)	**0.001 ^b^**
**Polytrauma (yes)**	13 (3.3%)	0 (0.0%)	**0.024 ^c^**
**Completeness of Injury (ASIA Scale)**			
A	181 (45.0%)	19 (12.0%)	**<0.001 ^d^**
B	78 (19.4%)	20 (12.7%)	
C	114 (28.4%)	96 (60.8%)	
D	29 (7.2%)	23 (14.6%)	
**Neurological level of injury**			
Cervical	131 (32.6%)	58 (36.7%)	**0.006 ^b^**
Thoracic	155 (38.6%)	39 (24.7%)	
Lumbar	116 (28.9%)	61 (38.6%)	
**Length of stay (days)**	164.8 ± 89.5	95.9 ± 66.9	**<0.001 ^a^**

All values presented as n (%) or mean ± SD. *p*-value < 0.05 was considered statistically significant. Tests: ^a^ *t*-test, ^b^ Chi-squared, ^c^ Fischer’s Exact Test, ^d^ Mann–Whitney U.

**Table 2:** The frequency of urinary tract infections in relation to the manner of bladder emptying.

	UTI		
	Yes (n = 402)	No (n = 158)	*p*-Value
Spontaneous	24 (6.0%)	115 (72.8%)	**<0.001**
Intermittent self-catheterization	252 (62.7%)	35 (22.2%)	
Intermittent catheterization	69 (17.2%)	3 (1.9%)	
Reflex emptying	26 (6.5%)	3 (1.9%)	
Indwelling catheter	31 (7.7%)	2 (1.3%)	

All values presented as n (%). Chi-squared test. *p*-value < 0.05 was considered statistically significant.

**Table 3:** Frequency of UTIs by bladder dysfunction.

	UTI		
	Yes (n = 402)	No (n = 158)	*p*-Value
No bladder dysfunction	21 (5.2%)	116 (73.4%)	**<0.001**
Hyper-reflexive bladder	200 (49.8%)	12 (7.6%)	
Hypotonic bladder	181 (45.0%)	30 (19.0%)	

All values presented as n (%). Chi-squared test. *p*-value < 0.05 was statistically significant.

**Table 4:** Univariate analysis and multivariate logistic regression analysis.

	Univariate			Multivariate		
	B	*p*-Value	OR (95%CI)	B	*p*-Value	aOR (95%CI)
**Age**	−0.023	**<0.001**	0.98 (0.97–0.99)	0.007	0.451	1.00 (0.99–1.03)
**Gender (F vs. M)**	−0.227	0.269	0.80 (0.53–1.19)	0.549	0.099	1.73 (0.90–3.32)
**Etiology (traumatic vs. non-traumatic)**	1.279	**<0.001**	3.59 (2.45–5.28)	0.474	0.130	1.61 (0.87–2.97)
**Type of lesion (complete vs. incomplete)**	1.666	**<0.001**	5.29 (3.21–8.72)	0.155	0.679	1.17 (0.56–2.44)
**Calculus during rehabilitation**	1.407	0.060	4.08 (0.94–17.68)	1.562	0.214	4.77 (0.41–56.09)
**Neurological level of injury**						
Cervical	Reference			Reference		
Thoracic	0.565	**0.018**	1.76 (1.10–2.81)	−0.049	0.883	1.06 (0.48–2.33)
Lumbar	−0.172	0.441	0.84 (0.54–1.30)	−0.703	0.304	0.67 (0.31–1.44)
**Type of bladder dysfunction**						
No bladder dysfunction	Reference			Reference		
Hyper-reflexive bladder	4.522	**<0.001**	92.06 (43.70–198.97)	4.266	**<0.001**	63.69 (28.39–142.91)
Hypotonic bladder	3.506	**<0.001**	33.33 (18.21–60.99)	3.573	**<0.001**	15.92 (15.92–65.80)
**Manner of bladder emptying**						
Spontaneous	Reference					
Intermittent self-catheterization	3.541	**<0.001**	34.5 (19.62–60.66)			
Intermittent catheterization	4.702	**<0.001**	110.21 (32.00–379.61)			
Reflex emptying	3.726	**<0.001**	41.53 (11.62–148.39)			
Permanent catheter	4.308	**<0.001**	74.27 (16.64–331.54)			
**Length of stay (days)**	0.012	**<0.001**	1.01 (1.009–1.015)	0.008	**0.002**	1.008 (1.004–1.012)

aOR-adjusted odds ratio; CI-confidence interval; OR-odds ratio. *p*-value < 0.05 was statistically significant.

## Data Availability

The data presented in this study are available on request from the corresponding author due to privacy reasons.
